# Testing Wearable UV Sensors to Improve Sun Protection in Young Adults at an Outdoor Festival: Field Study

**DOI:** 10.2196/21243

**Published:** 2020-09-16

**Authors:** Caitlin Horsham, Jodie Antrobus, Catherine M Olsen, Helen Ford, David Abernethy, Elke Hacker

**Affiliations:** 1 School of Public Health and Social Work Queensland University of Technology Brisbane Australia; 2 Preventive Health Branch Queensland Health Queensland Government Brisbane Australia; 3 Population Health Department QIMR Berghofer Medical Research Institute Brisbane Australia

**Keywords:** melanoma, health promotion, public health, preventive medicine, sunlight, sunburn, adolescents

## Abstract

**Background:**

Australia and New Zealand have the highest skin cancer incidence rates worldwide, and sun exposure is the main risk factor for developing skin cancer. Sun exposure during childhood and adolescence is a critical factor in developing skin cancer later in life.

**Objective:**

This study aims to test the effectiveness of wearable UV sensors to increase sun protection habits (SPH) and prevent sunburn in adolescents.

**Methods:**

During the weeklong school leavers outdoor festival (November 2019) at the Gold Coast, Australia, registered attendees aged 15-19 years were recruited into the field study. Participants were provided with a wearable UV sensor and free sunscreen. The primary outcome was sun exposure practices using the SPH index. Secondary outcomes were self-reported sunburns, sunscreen use, and satisfaction with the wearable UV sensor.

**Results:**

A total of 663 participants were enrolled in the study, and complete data were available for 188 participants (188/663, 28.4% response rate). Participants provided with a wearable UV sensor significantly improved their use of sunglasses (*P*=.004) and sunscreen use both on the face (*P*<.001) and on other parts of the body (*P*=.005). However, the use of long-sleeve shirts (*P*<.001) and the use of a hat (*P*<.001) decreased. During the study period, 31.4% (59/188) of the participants reported receiving one or more sunburns. Satisfaction with the wearable UV sensor was high, with 73.4% (138/188) of participants reporting the UV sensor was helpful to remind them to use sun protection.

**Conclusions:**

Devices that target health behaviors when outdoors, such as wearable UV sensors, may improve use of sunscreen and sunglasses in adolescents.

## Introduction

Skin cancer is Australia’s national cancer and was estimated to account for more cases diagnosed than all other cancers combined and costing over US $580 million to treat each year [1-3]. Melanoma is the deadliest form of skin cancer and the most commonly diagnosed cancer in young adults aged 15-29 years in Australia [4]. Sunlight or ultraviolet radiation (UVR) is the main risk factor for skin cancer. Childhood and adolescence are critical periods during which exposure to UVR contributes to skin cancer in later life [5]. The amount of sun exposure received during this period is high, with half of our total lifetime sun exposure received within the first 20 years [6]. Sunburn remains highly prevalent in Australia in the younger age groups, and as little as one severe sunburn in childhood can double the risk of developing a melanoma before the age of 40 years [6,7]. Australian adolescents continue to report high-risk behaviors such as spending long periods of time in the sun and positive views of tanning [8]. These findings are concerning and highlight the importance of preventative strategies during adolescence or young adult years, which may have a substantial impact on health outcomes later in life [9].

Daily sunscreen use at the population level has been shown to prevent keratinocyte cancers and melanoma deaths and to reduce health care costs [10]. Although adolescents have been reported to be knowledgeable about the risks of UVR and skin cancer, this knowledge does not always equate with the use of sunscreen or other sun protection practices [11,12]. Since the 1980s, mass media campaigns have been circulated in Australia, the most well-known being Slip, Slop, Slap, to raise awareness of the importance of sun protection [13]. To further motivate this hard-to-reach adolescent population, recent strategies have included social media campaigns, and positive impacts have been reported for the Sun Mum, Dear Melanoma, and Pretty Shady campaigns [14,15]. Technology including apps [16-18], UVR monitors or dosimeters [19], and UV detection stickers [20] can target people at the actual point of behavior as a *cue to action* [21]. The mobile phone app, Solar Cell, provided personalized time until sunburn information to over 600 US residents, aged >18 years, which led to a significant increase in sun protection behaviors [16]. Our research showed that young adults carrying a UVR dosimeter, which alarmed when UVR threshold levels were reached, reduced their time unprotected and exposed to UVR on weekends [19].

Outdoor festivals and mass gatherings of adolescents may pose certain health risks [22]. School leaver festivals are common events across Australia and are often referred to as *schoolies celebrations* to mark the graduation of students from the secondary education system. The Gold Coast school leavers festival is ticketed and incorporates free outdoor music concerts and beach activities in a high UVR environment and has been operating for over 10 years with between 16,000 and 18,000 registered attendees each year [23]. During festivals, some youth may engage in risky health behaviors, such as alcohol consumption or using illicit drugs [24]. Sunburn is also common at outdoor festivals in Australia when people are exposed to UVR during peak hours for long periods of time, and low adherence to the use of sun protective clothing is frequently observed [25]. An observational study reported that 14% of event attendees wore long-sleeve shirts, 56% wore hats, and 83% wore sunglasses [25]. In Australia, festival and event organizers are responsible for providing a safe environment for all attendees and staff [26]. Implementation of harm minimization strategies, including effective preplanning and resource provisions, is necessary to produce a safe environment. At the school leavers festival on the Gold Coast, all attendees must wear a wristband to gain entry to events. The addition of a sun safety prompt to this wristband could provide tailored health information to each user as a call to action, with feedback provided when it has the most potential to be beneficial during UVR exposure [21].

This research describes the development of a wearable UV sensor and presents the findings of a field study testing if the device could improve sun protection behaviors and reduce sunburn among adolescents at a weeklong outdoor school leavers festival.

## Methods

### Field Study Participants and Setting

Participants in the field study were attending the weeklong school leavers outdoor festival at the Surfers Paradise beach on the Gold Coast (latitude 28^o^S, 153^o^E) during November 16-22, 2019 (spring) in Australia.

The field study was approved by the Human Research Ethics Committee of the Queensland University of Technology (QUT; #1900000435) and prospectively registered with the Australian and New Zealand clinical trials register (ACTRN12619000976189).

### Wearable UV Sensor Development

The wearable UV sensor was developed by the researchers for the school leavers outdoor festival in collaboration with the event organizers. The UV sensor was fitted to the event wristband and was developed to change color when exposed to sunlight. The UV sensor component was developed using a UV-responsive photochromic dye and molded into a silicon slider to fit over the existing wristband fabric. The event wristband is designed not to be removed once worn, and the fabric material has a locking mechanism that requires the wristband to be cut for removal. On the UV sensor, the slogan *be safe and watch your mates* was added as a reminder to avoid risky situations during the festival. The measurement accuracy of the silicon slider was determined using a UV intensity meter (Solar Light Co, Model PMA2100) fitted with a digital sensor (Solar Light Co, model PMA2101). Prototype and safety testing were undertaken in Brisbane (latitude 27^o^S, 153^o^E) during May (autumn) in Australia (Multimedia Appendix 1).

### Field Study Recruitment and Treatment Regimen

At the start of the outdoor festival, school leavers attended a registration event where participants were recruited, completed a baseline questionnaire, and were provided with their choice of a free tube of sunscreen (Cancer Council SPF 50+, 75 mL or 110 mL) for use during the festival. All individuals attending the school leavers outdoor festival were provided with the wearable UV sensor regardless of their participation in the study. The wearable UV sensor was required to be worn by the festival attendees as a wristband for the duration of the festival to gain entry to the festival events. During the registration process, event staff secure the wristband to the attendees’ wrist. If the wristband is removed during the festival, participants are required to obtain a replacement wristband to gain entry to events. Posters displayed at the registration event described how the wearable UV sensors functioned (Multimedia Appendix 1). A follow-up web-based survey was emailed 7 days after the participant attended the outdoor festival, which asked about their sun protection behaviors during the festival (November 17-22, 2019). The follow-up survey did not collect sun protection behavior data for the first day of the festival, when participants enrolled in the study and collected their intervention device. Data collection was managed using REDCap electronic data capture tools hosted at the QUT, and surveys are shown in Multimedia Appendix 2 [27]. Participants received an initial email initiation to complete the follow-up survey on the web, followed by two reminders.

### Weather Measurements During the Field Study

UVR data were recorded using a UV-Biometer model 501 (Solar Light Co), and data were displayed using the UV index scale. The standard erythemal dose (SED) was also calculated using daily summaries and hourly observations recorded at 10 AM and at noon. The UVR data were captured by the Australian Radiation Protection and Nuclear Safety Agency detector (Gold Coast, latitude 28^o^S, 153^o^E).

The proportion of cloud cover in the sky above the Surfers Paradise beach was recorded twice a day in the morning between 8 AM and 10 AM and in the afternoon between 2 PM and 4 PM. Images of the sky above the beach were captured using a fixed camera maintained by Coastalwatch [28]. The proportion of cloud cover in each image was counted using ImageJ software [29], as described previously [20]. All field trial image analysis and quantification procedures were performed blind to the image ID.

Temperature data were recorded in degrees Celsius, and data were reported for the daily minimum and maximum as well as observations at 9 AM and 3 PM each day. The temperature data were captured by the Bureau of Meteorology weather station (040764; Gold Coast Seaway, latitude 28^o^S, 153^o^E).

### Outcome Measures

The primary outcome measure was the sun protection habits (SPH) index described by Glanz et al [30] and updated by Heckman et al [31] for young adults, which queries the frequency of 7 sun protective habits that are used when outdoors using a 4-point Likert scale (1=never or rarely, 2=sometimes, 3=usually, and 4=always), which are averaged to derive the score, including wearing a shirt with sleeves, wearing a hat, wearing sunglasses, wearing sunscreen with a sun protection factor (SPF) 15 or higher on the face, wearing sunscreen with an SPF 15 or higher on other parts of the body, staying in the shade, and limiting time in the sun during midday hours. Secondary outcomes were self-reported, including the number of sunburns; sunburn intensity (mild, moderate, or severe) and location of sunburn; sunscreen use, including the frequency of daily application; and satisfaction with the wearable UV sensor. In the follow-up survey, participants completed an open-answer question on their comments or suggestions about the study.

### Statistical Analysis

The Pearson chi-squared and/or Fisher exact test was used to detect the statistical significance in the difference between the groups who completed the follow-up survey and those who did not. For the participants who completed the follow-up survey, the Wilcoxon matched-pairs signed-rank test was used to examine the differences in sun protection behaviors at baseline and at follow-up. Changes in SPH were measured, and participants were grouped by comparing an individual’s baseline value with their follow-up value and allocating them to either the improved, decreased, or no change groups. Analyses were performed using SAS statistical software package (SAS Institute).

Inductive thematic analysis was used to group open-ended answers into themes by 2 researchers (CH and DA).

## Results

### Wearable UV Sensor Observational Testing

**Figure 1:**
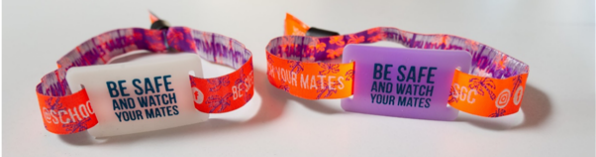
Wearable UV sensor. The wearable UV sensor is white (left image) which indicates no UV exposure. The UV indicator is purple (right image) demonstrating exposure to UV radiation.

The wearable UV sensor comprises a UV-responsive photochromic dye that is white when not in sunlight and turns purple in sunlight, indicating that sun protection is required (Figure 1). The threshold for the color change was tested using a graded series of UVR intensities. The color change was observed in part shade when the UVR level was low, 0.21 uW/cm^2^, and no color change was observed when the indicator was in full shade, 0.0 uW/cm^2^. When the sensor was placed in the sun (UVR=5.27 uW/cm^2^), the sensor immediately changed color to purple (Multimedia Appendix 1). The photochromic dye in the wearable UV sensor was shown to be responsive to low-level UVR intensity, demonstrating that it was an adequate indicator for use in the field study.

### Field Study Participant Characteristics

**Figure 2:**
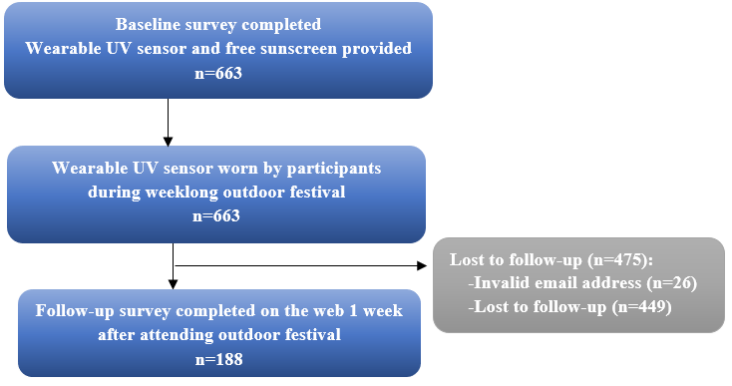
During recruitment participants completed either a written or web-based baseline survey, and some email addresses were not completed correctly; invalid email address=email bounced back and the follow-up survey was not received by the participant.

During the first day of the festival, 663 volunteers were enrolled and completed the baseline survey. The evaluation survey was completed by 188 (28.4%) participants (Table 1; Figure 2). There were demographic differences between those who completed the study (n=188) and those who did not (n=475), with females and people with brown hair more likely to complete the study (Table 1).

Of the 188 participants who completed the study, most participants had very fair or fair skin (114/188, 60.6%), were women (145/188, 77.1%), and their age ranged from 15 to 19 years (Table 1).

**Table 1:** Field study participant characteristics.

Characteristics		Baseline (n=663)	No evaluation; (n=475)	Evaluation (n=188)		*P* value
Age (years), range		15-19	17-19	15-18		.12
**Gender, n (%)**					.006	
	Female	452 (68.2)	307 (64.6)	145 (77.1)		
	Male	208 (31.4)	165 (34.7)	43 (22.9)		
	Other	3 (0.5)	3 (0.6)	0 (0)		
**Skin color, n (%)**					.25	
	Very fair or fair	379 (57.2)	265 (55.8)	114 (60.6)		
	Medium	186 (28.1)	139 (74.0)	47 (25.0)		
	Olive or brown	97 (14.6)	71 (15.0)	26 (13.8)		
	Missing	1 (0.2)	0 (0)	1 (0.5)		
**Hair color, n (%)**					.008	
	Red (including auburn)	54 (8.1)	40 (8.4)	14 (7.4)		
	Fair or blonde (including white)	154 (23.2)	126 (26.5)	28 (14.9)		
	Light brown, mouse brown, or dark brown	418 (63.1)	282 (59.4)	136 (72.3)		
	Black	36 (5.4)	27 (5.7)	9 (4.8)		
	Missing	1 (0.2)	0 (0)	1 (0.5)		
**Skin burn in strong summer sun for 30 min without protection?, n (%)**					.15	
	My skin would not burn at all	99 (14.9)	78 (16.4)	21 (11.2)		
	My skin would burn lightly	286 (43.1)	203 (42.7)	83 (44.2)		
	My skin would burn moderately	228 (34.4)	163 (34.3)	65 (34.6)		
	My skin would burn severely	49 (7.4)	31 (6.5)	18 (9.6)		
	Missing	1 (0.2)		1 (0.5)		
**Skin tan in strong sun without protection?, n (%)**					.17	
	My skin would not tan	122 (18.4)	79 (16.6)	43 (22.9)		
	My skin would tan lightly	224 (33.8)	164 (34.5)	60 (31.9)		
	My skin would tan moderately	233 (35.1)	175 (36.8)	58 (30.9)		
	My skin would tan deeply	84 (12.7)	57 (12.0)	27 (14.4)		
**Have you made an attempt to get a suntan in the last 12 months?, n (%)**					.49	
	Yes	435 (65.6)	317 (66.7)	118 (62.8)		
	No	227 (34.2)	157 (33.1)	70 (37.2)		
	Missing	1 (0.2)	1 (0.2)	0 (0)		
**During the past 12 months, how many times did you get sunburnt?, n (%)**					.31	
	Never	43 (6.5)	34 (7.2)	9 (4.8)		
	Once	138 (20.8)	102 (21.5)	36 (19.1)		
	2-5 times	349 (52.6)	239 (50.3)	110 (58.5)		
	≥6 times	97 (14.6)	71 (15.0)	26 (13.8)		
	Do not know or unsure	36 (5.5)	29 (6.1)	7 (3.7)		

### Sun Protection Items Brought to the Festival by Participants

Sun protection items were common among all participants (n=663), with 76.6% (508/663) bringing a hat, 76.0% (504/663) bringing sunglasses, and 66.0% (438/663) bringing sunscreen, whereas only 41.6% (276/663) brought a long-sleeve shirt and 12.2% (81/663) brought a beach umbrella (Multimedia Appendix 2).

### Weather Conditions During the Field Study

The UVR exposure level was consistently high, requiring sun protection each day during the weeklong outdoor festival with daily SEDs ranging from 36 SEDs to 54 SEDs (Table 2). The UV index level was >3 for over 6 hours each day of the festival, the cloud cover was consistently clear throughout the week, and there was no rainfall recorded (Table 2). The average daily maximum temperature was 28°C (range 26.1-30.1) during the field study (Table 2).

**Table 2:** Weather conditions during the field study.

Date	Start of day when UVI^a^ >3, AM	End of day when UVI <3, PM	Temperature, °C				UV daily^b^ dose	UV 10-11 AM^c^ dose	UV noon -1 PM^d^ dose	Cloud cover	Rain (mm)
			Minimum	Maximum	9 AM	3 PM	SEDs^e^	SEDs	SEDs		
November 16, 2019	8	3	21.5	30.1	28.7	26.1	47	8	8	Nil	0
November 17, 2019	8	2	19.9	27.6	24.4	23.9	42	7	8	Nil	0
November 18, 2019	8:30	3:30	19.5	26.1	23.5	23.9	54	9	9	Nil	0
November 19, 2019	8:30	2:30	17.7	29.2	25.0	25.6	36	7	6	Nil	0
November 20, 2019	8:30	2:30	22.1	29.5	27.0	26.9	43	7	7	Nil	0
November 21, 2019	8:30	3	21.7	27.4	25.7	25.3	47	7	8	Nil	0
November 22, 2019	8:30	3	19.9	27.0	24.8	25.5	42	7	6	Nil	0

^a^UVI: ultraviolet index.

^b^Daily dose calculated from 6 AM-4 PM.

^c^Morning dose calculated from 10 AM-11 AM.

^d^Midday dose calculated from noon-1 PM.

^e^SEDs: standard erythemal dose.

### SPH Index

At baseline (n=188), the mean SPH index value was 2.31 (SE 0.04) and did not change at follow-up (+0.03; Table 3). Some individual SPH items improved significantly at follow-up compared with baseline, including use of sunglasses (+0.21; *P*=.004), sunscreen use on the face (+0.36; *P*<.001), and sunscreen use on the body (+0.22; *P*=.005). The use of long-sleeve shirts (−0.31; *P*<.001) and use of a hat (−0.30; *P*<.001) both decreased significantly at follow-up.

Over 41.4% (78/188) of participants improved their Likert scale level from baseline for their use of sunscreen applied to the face, whereas 17.0% (32/188) of the participants decreased their usage and 41.5% (78/188) of the participants had no change at follow-up. Sunscreen applied to the body also improved, with 38.3% (72/188) of participants improving their Likert scale score, whereas 20.7% (39/188) of the participants decreased their usage and 40.9% (77/188) of the participants had no change at follow-up.

**Table 3:** Sun protection habits index.

Items		Baseline (n=188), mean (SE)	Follow-up (n=188), mean (SE)	Change	*P* value^a^
**SPH^b^ index (below items combined)**		2.31 (0.04)	2.34 (0.03)	+0.03	No change
	Wear a shirt with long sleeves	1.66 (0.05)	1.35 (0.05)	−0.31	<.001
	Wear sunglasses	2.30 (0.07)	2.51 (0.08)	+0.21	.004
	Stay in the shade	2.50 (0.03)	2.60 (0.05)	+0.10	.31
	Limit your time in the sun during midday hours	2.40 (0.06)	2.30 (0.06)	−0.10	.48
	Wear a hat	2.41 (0.07)	2.11 (0.07)	−0.30	<.001
	Wear sunscreen with an SPF^c^ ≥15 on your face	2.46 (0.06)	2.82 (0.07)	+0.36	<.001
	Wear sunscreen with an SPF^c^ ≥15 on other parts of your body	2.43 (0.06)	2.65 (0.06)	+0.22	.005

^a^Wilcoxon matched-pairs signed rank test.

^b^SPH: sun protection habits. 1=never or rarely, 2=sometimes, 3=usually, and 4=always.

^c^SPF: sun protection factor.

### Sunburn, Sunscreen Usage, and Time Outdoors

The baseline survey included a question about sunburns during the week before the festival, and 36.7% (69/188) of the participants reported one or more sunburns. During the festival, 31.4% (59/188) of the participants reported being sunburnt (Table 4). During the 12 months before the festival, over 91.4% (172/188) of the participants reported one or more sunburns (Table 1).

During the outdoor festival, sunburns were most commonly reported on the shoulders (36/109, 33.0%), followed by the head or face (27/109, 24.8%), neck (19/109, 17.4%), and legs (10/109, 9.2%; Table 4). The majority of sunburns reported were of mild intensity (71/109, 65.1%), followed by moderate (32/109, 29.4%) and severe (4/109, 3.7%; Table 4).

Participants who reported seeking a deliberate suntan in the previous year were more likely to report a sunburn during the outdoor festival (75% vs 57%; *P*=.02; Multimedia Appendix 2). Participants who reported a sunburn were also more likely to have not worn the wearable UV sensor (9% vs 2%; *P*=.05; Multimedia Appendix 2).

Sunscreen use was commonly reported in the follow-up survey, with 88.3% (166/188) of participants applying sunscreen one or more times during the day at the outdoor festival (Table 4). Most participants who reported being sunburned also reported applying sunscreen (51/59, 86%), and just under half of those sunburnt reported reapplying sunscreen two or more times per day (27/59, 46%).

Most participants (115/188, 61.2%) spent 2 hours outside each day of the festival; 30.8% (58/188) of the participants spent 1-2 hours outdoors and only 7.9% (15/188) spent ≤1 hour outdoors each day.

**Table 4:** Sunburn, sun tanning, and sunscreen use.

Survey items		Baseline (N=188), n (%)	Follow-up (N=188), n (%)
**Did you experience any sunburn?^a^**			
	Yes	69 (36.7)	59 (31.4)
	No	119 (63.3)	129 (68.6)
Total number of sunburn events^b^		142	109
**Location of sunburns**			
	Body locations below combined	142 (100)	109 (100)
	Head or face	50 (35.2)	27 (24.8)
	Neck	20 (14.1)	19 (17.4)
	Shoulders	30 (21.1)	36 (33.0)
	Back	10 (7.1)	4 (3.7)
	Chest	1 (0.7)	0 (0)
	Arms	14 (9.9)	6 (5.5)
	Hands	3 (2.1)	1 (0.9)
	Legs	7 (4.9)	10 (9.2)
	Feet	7 (4.9)	5 (4.6)
	Buttocks	0 (0)	1 (0.9)
**Intensity of sunburns**			
	Mild (pink to light redness)	64 (45.1)	71 (65.1)
	Moderate (red skin)	66 (46.5)	32 (29.4)
	Severe (deep redness, blisters may develop)	5 (3.5)	4 (3.7)
	Missing	7 (4.9)	2 (1.8)
**Have you tried to get a suntan during the festival?**			
	Yes	N/A^c^	97 (51.6)
	No	N/A	91 (48.4)
**Number of sunscreen applications per day during the festival**			
	0	N/A	22 (11.7)
	1	N/A	86 (45.7)
	≥2	N/A	80 (42.6)

^a^Baseline data collection: During the past week, how many times did you get sunburnt? Follow-up data collection: “We would like to know if you experienced any sunburn during November 17th to November 22nd 2019?”

^b^Incidence of sunburnt body areas. Participants may have received multiple sunburn events.

^c^N/A: not applicable.

### Satisfaction With the Wearable UV Sensor

Adherence to the intervention device was high, with 95.7% (180/188) of the participants wearing the wearable UV sensor during the outdoor festival (Multimedia Appendix 2). Over 73.4% (138/188) of the participants found the wearable UV sensor helpful to remind them to apply sunscreen, whereas 16.5% (31/188) reported that it was not helpful and 10.1% (19/188) were unsure. Over 83.5% (157/188) of the participants would like to have this product included on wristbands for future daytime outdoor festivals. On a scale from 1 (not at all satisfied) to 10 (extremely satisfied), participants’ mean satisfaction score was high at 8.1 (range 2-10; Multimedia Appendix 2).

Open-ended questions were completed by 50.0% (94/188) of the participants. The themes discussed included helpful, reinforced behaviors, recommendations for improvements, lack of awareness, enjoyment, and lack of impact and appearance. A participant commented that the wristband and embedded UV sensor was fashionable “I thought it was a cool item that wasn’t overly apparent, so it went with the things you were wearing.” The description of each theme along with example comments from participants are shown in Multimedia Appendix 2.

## Discussion

### Principal Findings

In this study we developed a wearable sun safety device and investigated the impact of the device to improve adolescents’ use of sun protection behaviors at an outdoor festival. We found that providing participants with a wearable UV sensor improved their use of individual SPH items, including use of sunscreen and sunglasses. There was no improvement in the combined SPH index as a result of participants decreasing their use of long-sleeve shirts, along with the use of a hat. There was no significant change in the use of shade or time spent outdoors, which is not surprising given the outdoor festival context. Only under one-third of the participants experienced a sunburn during the outdoor festival, with the shoulders most commonly sunburnt. Our results indicate that participants who had a history of deliberate suntanning were significantly more likely to receive a sunburn during the outdoor festival. Participants reported high satisfaction rates, with most participants reporting that they would like to have the wearable UV sensor available at future outdoor events.

Sun protection is a multifaceted behavior in adolescents, involving a wide range of factors. In this field study, participants who exhibited high-risk behaviors, such as deliberate tanning, were more likely to receive a sunburn. A previous study of 4150 adolescents aged 14-17 years showed this association between adolescent sunburn and a positive attitude toward tanning as well as high rates of tanning, with 48% reporting they liked to tan [32]. The desire for tanned skin has been reported as a significant barrier to using sun protection in adolescents, with a tan associated with providing a sense of confidence, achievement, attractiveness, and the ability to fit in with their peers [8]. A photoaging app, called Sunface, which predicts the effects of sun damage to the face (such as wrinkles or aging) was tested in adolescents and was found to be effective in changing tanning and sun safe behaviors [33]. During 2010-2014, the nationwide campaign *The dark side of tanning* targeted young Australians’ attitudes toward tanning and was shown to be effective at reducing positive attitudes toward tanning [34]. In Australia’s nationwide sun protection telephone survey, 60% of adolescents reported a preference to tan in 2003, which dropped to 38% in 2013, and remained unchanged in 2019 [35]. A review of interventions to reduce tanning has shown that many programs are designed to increase knowledge of the risks of tanning and that there is the potential to formulate new programs to incorporate relevant addiction science techniques, including the use of brief motivational and cognitive behavioral-based interventions [36]. Tanning behavior is complex and multifaceted, and interventions are needed to address this issue as a high proportion of adolescents are still partaking in this high-risk behavior.

In adolescents, clothing choices are mostly influenced by fashion trends rather than sun protection [8]. A survey of young female beachgoers aged 17-35 years in Australia found that their sun protection at the beach was influenced by being uncomfortable or unstylish and whether friends or peers approved of their sun safe behavior [37]. In our study, sun protective clothing such as long-sleeve shirts or hats did not improve during the festival, whereas the use of sunglasses did. The use of long-sleeved shirts may not be considered fashionable or practical in hot environments, and *hat hair* after using a hat, which can negatively alter a hairstyle, has previously been cited as a barrier to hat use in adolescents [8]. To effectively target sun protection behavior in youth culture, further interventions may need to focus on changing adolescents’ perceptions of what is healthy and fashionable [38].

A key priority for skin cancer prevention technology should be to understand the factors that reduce the incidence of sunburn. In our study, 31% of the participants reported one or more sunburns during the festival, which is similar to previous estimates showing 26% of Australian adolescents aged 12-17 years reported being sunburnt on summer weekends [39]. Many participants in this study reporting sunburns also reported wearing and reapplying sunscreen, suggesting sunscreen was not applied sufficiently to provide adequate UV protection. The reasons that people may receive an unintended sunburn after sunscreen use has been explored using qualitative interviews, which showed adults overestimated the amount of time they could safely be exposed to the sun and not reapplying sunscreen often enough, especially during water-based activities [40]. This study was based on a beach environment that may involve swimming, and this requires more frequent sunscreen applications. The wearable UV sensor was worn by almost all participants for the duration of the study, highlighting the robustness of the device.

Another key barrier to sun protection reported by adolescents is forgetting to protect their skin [41,42]. This may explain why adolescents have good sun protection knowledge but still report high rates of sunburn. To reduce the impact of forgetfulness, interventions that act as a reminder to target behaviors at the actual point of need are required [21]. The wearable UV sensor developed in this study, which changes color in the sun notifying the user that sun protection is required, is an example of a real-time, wearable prompt for sun safety. Other technologies such as UV detection stickers have been shown to be useful prompts for the re-application of sunscreen. Our research previously tested UV detection stickers in 428 adults during an outdoor sporting event with the aim of improving the re-application of sunscreen [20]. We found that participants provided with a UV detection sticker were more likely to re-apply sunscreen than controls (80% vs 68%; *P*=.04); however, the stickers did not reduce sunburn rates [20]. The wearable UV sensors tested in this study notified the user when they were exposed to UVR, whereas UV detection stickers prompt the user when their sunscreen required re-application. These ecological momentary interventions are examples of reminders to influence behaviors within an environmental context, as tailoring the content of health messages based on individual characteristics can improve willingness to change [21].

### Strengths and Limitations

The strengths of this study include recruiting a large sample of adolescents who wore a UV sensor for 1 week in a high UV setting. This target group is typically difficult to reach in public health research, and the findings of this study could be applied to other high UV environments such as sporting events or summer festivals. Limitations of this study include self-reported outcome measures, including wearable UV sensor compliance as well as the low response rate for the follow-up survey, and no datasets were collected on long-term sustained behavior change. The wearable UV sensor developed in this study was designed to not be removed once worn and was required to be viewed each day for festival entry. Only 4.3% (8/188) of the participants in our study reported removing the UV sensor wristband and not wearing it during the outdoor festival, and we did not collect further data on if they obtained a replacement band. The Hawthorne effect, where study participants alter their behavior because of being observed, could be a potential factor for the increase in sunscreen use [43]. The extent to which the provision of free sunscreen contributed to behavior change compared with wearing the UV sensor remains unknown. However, in Australia, legislation states that all people who are exposed to sunlight at outdoor events should be able to re-apply sunscreen every 2 hours. Guidelines further stipulate that if security measures prevent sunscreen from being brought inside the venue, event organizers have a duty of care to provide easy access within the venue. In Australia, it is common for sunscreen to be promoted and supplied by event organizers at outdoor mass gathering as standard care.

Participants who completed the study were mainly young females, and the results may not be generalizable to other subgroups of the population. However, adolescents are commonly underrepresented in prevention studies, spend long periods of time in the sun, and have higher rates of sunburn than adults [44]. Selection bias was a further limitation as participants were recruited at this event using a sequential, convenience sample, and we did not use a random sampling method.

### Conclusions

This study developed and tested the effectiveness of a wearable UV sensor to improve sun protection and decrease sunburn in adolescents at an outdoor festival. Provision of a wearable UV sensor and free sunscreen improved use of sunglasses and sunscreen in participants. The wearable UV sensors did not reduce sunburn rates, and those who reported a history of suntanning were more likely to be sunburnt. The wearable UV sensor technology resonated with adolescent participants in this study with high satisfaction rates, and participants found them to be a helpful reminder for sun protection.

## Appendix

Multimedia Appendix 1 Supplementary figures.

Multimedia Appendix 2 Supplementary tables.

## Abbreviations

QUT: Queensland University of Technology
SEDs: standard erythemal doses
SPF: sun protection factor
SPH: sun protection habits
UVR: ultraviolet radiation
