# Supplementary material for: Testing Wearable UV Sensors to Improve Sun Protection in Young Adults at an Outdoor Festival: Field Study
Source: JMIR Mhealth Uhealth. 2020 Sep 16;8(9):e21243. doi: 10.2196/21243 (PMC7531871; doi:10.2196/21243)
Supplement: Multimedia Appendix 1 [file mhealth_v8i9e21243_app1.docx]

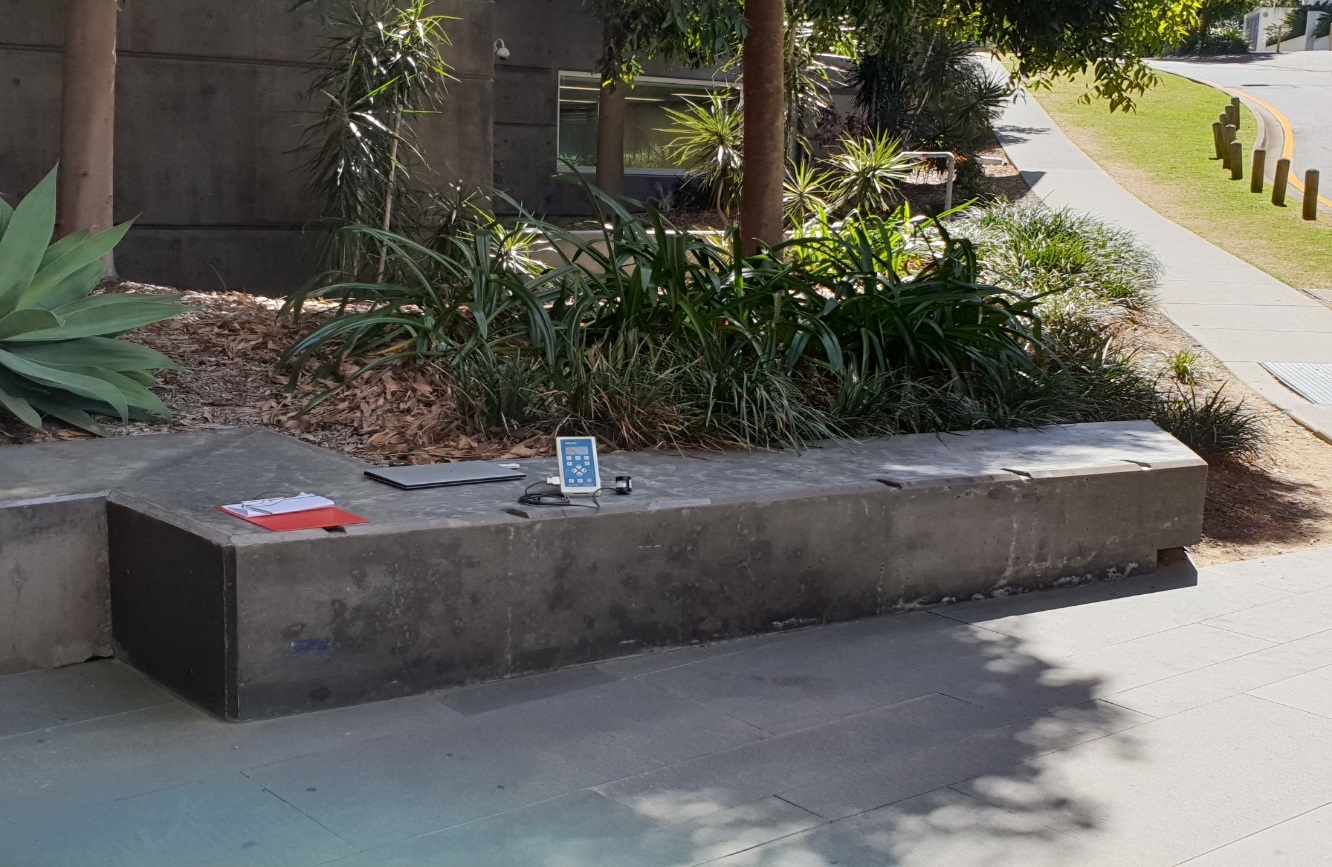


Full shade

Part shade

No shade

## Supplementary Figure 1. Measurement accuracy of the ultraviolet (UV) sensor along a gradient of UVR intensity and shade.


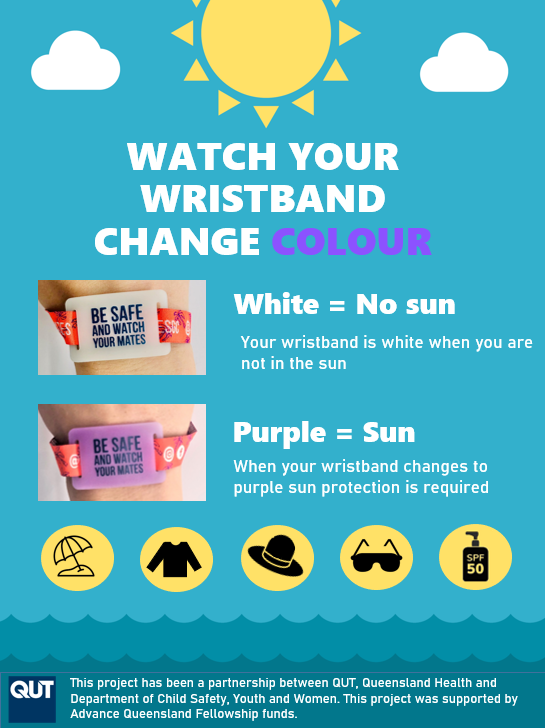


**Supplementary Figure 2. The function of the wearable UV sensor was shown using A3 posters displayed throughout the school leavers outdoor festival registration event.**
