# Supplementary material for: Testing Wearable UV Sensors to Improve Sun Protection in Young Adults at an Outdoor Festival: Field Study
Source: JMIR Mhealth Uhealth. 2020 Sep 16;8(9):e21243. doi: 10.2196/21243 (PMC7531871; doi:10.2196/21243)
Supplement: Multimedia Appendix 2 [file mhealth_v8i9e21243_app2.docx]

Supplementary Table 1. Structured Questionnaires used in the Study

| **Baseline Questionnaire** |
| --- |
| 1. Did you bring to Schoolies?  -Sunscreen  -A long-sleeve shirt  -Sunglasses  -A hat  -A beach umbrella |
| 2. Have you made an attempt to get a suntan in the past 12 months?  -Yes  -No |
| 3. During the past 12 months, how many times did you get sunburnt?  -Never (If no, go to Q.7)  -Once  -2-5 times  -6 or more times  -Don’t know/unsure (If don’t know/unsure, go to Q.7) |
| 4. If yes to Q.3., which body parts were sunburnt in the past 12 months? |
| Sunburn Intensity  -Mild (pink to light redness)  -Moderate (red skin)  -Severe (deep redness, blister may develop) |
| 5. During the past WEEK, how many times did you get sunburnt?  -Never (If no, go to Q.7)  -Once  -2-5 times  -6 or more times  -Don’t know/unsure (If don’t know/unsure, go to Q.7) |
| 6. If yes to Q.5., which body parts were sunburnt in the past WEEK?  Sunburn Intensity  -Mild (pink to light redness)  -Moderate (red skin)  -Severe (deep redness, blister may develop) |
| 7. In the past 3 months, please tell us how many HOURS you typically spent outdoors and in the sun EACH DAY…  ….MONDAY to FRIDAY   - 0-1 hours - 1-2 hours - 2-3 hours - 4 or more hours   ….SATURDAY to SUNDAY   - 0-1 hours - 1-2 hours - 2-3 hours - 4 or more hours |
| 8. When outdoors, how often do you regularly?  -Wear a shirt with long sleeves  - Wear sunglasses  - Stay in the shade  - Wear sunscreen with an SPF of 15 or more on your face  - Wear sunscreen with an SPF of 15 or more on other parts of your body  - Limit your time in the sun during midday hours  - Wear a hat |
| 9. What is your gender?  -Male  -Female  -Other |
| 10. How old are you? |
| 11. What is your skin colour before tanning or on areas never exposed to the sun, such as the inside of your upper arm?  - Very Fair  - Fair  - Medium  - Olive or brown |
| 12. Imagine that you were on the beach in the strong sun for 30 minutes in the middle of the day, without any protection such as sunscreen or clothing, for the first time in summer. How much would your skin burn?  - My skin would not burn at all  - My skin would burn lightly  - My skin would burn moderately  - My skin would burn severely |
| 13. Now, imagine you spend several weeks at the beach and you are often in the strong sun, without any protection such as sunscreen or clothing. How much would your skin tan?  -My skin would not tan  -My skin would tan lightly  -My skin would tan moderately  -My skin would tan deeply |
| 14. What is your NATURAL hair colour?  - Red (including auburn)  - Fair or blonde (including white)  - Light or mouse brown  - Dark brown  - Black |
|  |
| **Follow-up Questionnaire** |
| Have you made an attempt to get a suntan during November 17th to November 22^nd^ 2019?? |
| We would like to know if you experienced any sunburn during November 17^th^ to November 22^nd^ 2019?  - Yes  - No  If yes, how many times were you sunburnt? |
| If yes, which body part was sunburnt? |
| For the sunburn was it?  - Mild (pink to light redness)  - Moderate (red skin)  - Severe (deep redness, blisters may develop) |
| Did you use the sunscreen we gave to you? |
| On average, how many times did you apply sunscreen PER DAY during from November 17^th^ to November 22^nd^ 2019? |
| From November 17^th^ to November 22^nd^ 2019, please tell us how many HOURS you typically spent outdoors and in the sun EACH DAY…   - 0-1 hours - 1-2 hours - 2-3 hours - 4 or more hours |
| When you received the schoolies wristband did you wear it?  - Yes  - No |
| Did you need a reminder to help with applying and reapplying sunscreen?  - Yes  - No |
| Did you find the UV indicator on the wristband helpful to remind you to use sun protection?  - Yes  - No |
| Did you find yourself applying more sunscreen than you usually would?  - Yes  - No |
| From November 17^th^ to November 22^nd^ 2019, when outdoors, how often did you regularly?  -Wear a shirt with long sleeves  - Wear sunglasses  - Stay in the shade  - Wear sunscreen with an SPF of 15 or more on your face  - Wear sunscreen with an SPF of 15 or more on other parts of your body  - Limit your time in the sun during midday hours  - Wear a hat |
| Overall, how satisfied were you with the UV indicator on the wristband you received as part of the study? *(On a scale from 1, not at all satisfied to 10 Extremely Satisfied)* |
| Did you have any feedback about the UV indicator on the wristband? |
| Would you like to have this product included on wristbands for daytime outdoor festivals?  - Yes  - No |
| Did you have any other comments or suggestions about the study? |

## Supplementary Table 2. Sun Protection Items Brought to Festival by Participants

|  | **N = 663** | |
| --- | --- | --- |
|  | **n** | **(%)** |
| Sunscreen | | |
| Yes | 438 | (66.1) |
| No | 225 | (33.9) |
| A long sleeve shirt | | |
| Yes | 276 | (41.6) |
| No | 386 | (58.2) |
| Missing | 1 | (0.2) |
| Sunglasses | | |
| Yes | 504 | (76.0) |
| No | 157 | (23.7) |
| Missing | 2 | (0.3) |
| A hat | | |
| Yes | 508 | (76.6) |
| No | 154 | (23.2) |
| Missing | 1 | (0.2) |
| A beach umbrella | | |
| Yes | 81 | (12.2) |
| No | 581 | (87.6) |
| Missing | 1 | (0.2) |

## Supplementary Table 3. Participants who Received Sunburn During Festival

|  | Not sunburnt  N = 129 | | Sunburnt  N = 59 | | *p*-value |
| --- | --- | --- | --- | --- | --- |
|  | n | (%) | n | (%) |  |
| Gender | | | | | |
| Male | 27 | (20.9) | 16 | (27.1) |  |
| Female | 102 | (79.1) | 43 | (72.9) | 0.35 |
| Skin colour | | | | | |
| Very Fair/Fair | 71 | (55.0) | 43 | (72.9) |  |
| Medium | 38 | (29.5) | 9 | (15.3) |  |
| Olive/Brown | 19 | (14.7) | 7 | (11.9) |  |
| Missing | 1 | (0.8) | 0 | (0) | 0.11 |
| Hair colour | | | | | |
| Red/auburn/Blonde | 27 | (20.9) | 15 | (25.4) |  |
| Light brown/ mouse brown/Dark brown | 94 | (72.9) | 42 | (71.2) |  |
| Black | 7 | (5.4) | 2 | (3.4) |  |
| Missing | 1 | (0.8) | 0 | (0) | 0.76 |
| Attempted to get a suntan in the previous year | | | | | |
| Yes | 74 | (57.4) | 44 | (74.6) |  |
| No | 55 | (42.6) | 15 | (25.4) | 0.02 |
| Attempted to get a suntan during the festival | | | | | |
| Yes | 61 | (47.3) | 36 | (61.0) |  |
| No | 68 | (52.7) | 23 | (39.0) | 0.08 |
| On average, how many times per day did you apply sunscreen during the festival? | | | | | |
| 0 | 15 | (11.6) | 7 | (11.9) |  |
| 1 | 61 | (47.3) | 25 | (42.4) |  |
| 2-5 | 52 | (40.3) | 25 | (42.4) |  |
| More than 5 | 1 | (0.8) | 2 | (3.4) | 0.57 |
| Do you need a reminder to help with applying and reapplying sunscreen? | | | | | |
| Yes | 33 | (25.6) | 22 | (37.3) |  |
| No/unsure | 96 | (74.4) | 37 | (62.7) | 0.10 |
| When you received the wristband did you wear it? | | | | | |
| Yes | 126 | (97.7) | 54 | (91.5) |  |
| No | 3 | (2.3) | 5 | (8.5) | 0.05 |
| Did you find the wristband helpful to remind you to use sun protection? | | | | | |
| Yes | 90 | (69.8) | 48 | (81.4) |  |
| No/unsure | 39 | (30.2) | 11 | (18.6) | 0.10 |
| Did you find yourself applying more sunscreen than you usually would? | | | | | |
| Yes | 61 | (47.3) | 31 | (52.5) |  |
| No/unsure | 68 | (52.7) | 28 | (47.5) | 0.50 |

## Supplementary Table 4. Satisfaction with Wearable UV Sensor

|  | N = 188 | |
| --- | --- | --- |
|  | n | (%) |
| When you received the festival wristband did you wear it? | | |
| Yes | 180 | (95.7) |
| No | 8 | (4.3) |
| Did you find the wristband helpful to remind you to use sun protection? | | |
| Yes | 138 | (73.4) |
| No | 31 | (16.5) |
| Unsure | 19 | (10.1) |
| Did you find yourself applying more sunscreen than you usually would? | | |
| Yes | 92 | (49.0) |
| No | 79 | (42.0) |
| Unsure | 17 | (9.0) |
| Would you like to have this product included on wristbands for daytime festivals? | | |
| Yes | 157 | (83.5) |
| No | 9 | (4.8) |
| Unsure | 22 | (11.7) |
| Overall, how satisfied were you with the wristband you received as part of the study? *(On a scale from 1, not at all satisfied to 10 Extremely Satisfied.)* | | |
| Mean (range) | 8.1 (2-10) | |

Supplementary Table 5. Qualitative Analysis Themes, Theme Descriptions and Participant Responses

| **Theme** | **Frequency***  **(n)** | **Description** | **Responses** |
| --- | --- | --- | --- |
| Helpful | 25 | The wearable UV sensor was helpful, worked well and was a good reminder. | “It was very good, especially during the overcast days when the wristband would still change, indicating that UV rays are still prevalent.” |
| Reinforced Behaviours | 12 | The wearable UV sensor was educational and reinforced sun protection behaviours. | “It is a great idea and did help remind me to get out of the sun.” |
| Recommendations for Improvements | 23 | The size of the wearable UV sensor was too large, it did not record amount of UV exposure. | “The indicator was just a little too big and out there it could have been made smaller which I would have enjoyed more.” |
| Lack of Awareness | 6 | Not aware of the purpose or function of the wearable UV sensor. | “I don't fully understand the purpose of it at the start.” |
| Enjoyment | 46 | Descriptions of the wearable UV sensor as fun, cool, satisfying or a good idea. | “It was a fun way to be reminded.” |
| Lack of Impact | 7 | The wearable UV sensor did not change sun protection behaviours or the participant forgot about it. | “It was cool how it changed colour in the sun but I don't think it had a huge impact in terms of reminding me to put sunscreen on.” |
| Appearance | 5 | The aesthetics of the wearable UV sensor was both liked and disliked. | “they looked kinda ugly.”  “they looked great.”  “I liked the colours.” |

*number of comments received from participants relating to the theme
